# ProxyBind: A compendium of binding sites for proximity-induced pharmacology

**DOI:** 10.1016/j.csbj.2022.11.010

**Published:** 2022-11-08

**Authors:** Evianne Rovers, Lihua Liu, Matthieu Schapira

**Affiliations:** a Structural Genomics Consortium, University of Toronto, Toronto, ON M5G 1L7, Canada; b Department of Pharmacology and Toxicology, University of Toronto, Toronto, ON M5S 1A8, Canada

**Keywords:** PROTAC, Proteome, Proximity pharmacology, Kinases, Phosphatases, Post-translational modification, ProxPharm, Proximity Pharmacology, PROTAC, Proteolysis Targeting Chimera

## Abstract

Proximity-induced pharmacology (ProxPharm) is a novel paradigm in drug discovery where a small molecule brings two proteins in close proximity to elicit a signal, generally from one protein onto another. The potential of ProxPharm compounds as a new therapeutic modality is firmly established by proteolysis targeting chimeras (PROTACs) that bring an E3 ubiquitin ligase in proximity to a target protein to induce ubiquitination and subsequent degradation of the target. The concept can be expanded to induce other post-translational modifications via the recruitment of different types of protein-modifying enzymes. To survey the human proteome for opportunities in proximity pharmacology, we systematically mapped non-catalytic drug binding pockets on the structure of protein-modifying enzymes available from the Protein Databank. In addition to binding sites exploited by previously reported ProxPharm compounds, we identified putative ligandable non-catalytic pockets in 236 kinases, 45 phosphatases, 37 deubiquitinases, 14 methyltransferases, 11 acetyltransferases, 13 glycosyltransferases, 4 deacetylases, 7 demethylases and 2 glycosidases, including cavities occupied by chemical matter that may serve as starting points for future ProxPharm compounds. This systematic survey confirms that proximity pharmacology is a versatile modality with largely unexplored and promising potential and reveals novel opportunities to pharmacologically rewire molecular circuitries. All data is available from the ProxyBind database at https://polymorph.sgc.utoronto.ca/proxybind/index.php.

## Graphical abstract

**Figure:**
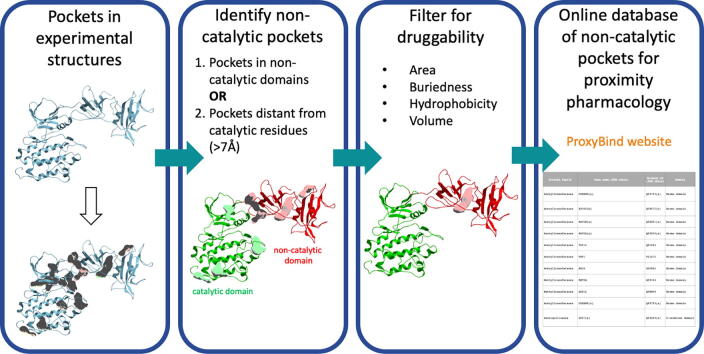

## 1 Introduction

Proteolysis targeting chimeras (PROTACs) are bifunctional small molecules that simultaneously bind an E3 ubiquitin ligase and a target protein, thereby inducing the ubiquitination and subsequent proteasomal degradation of the protein target [1]. This type of molecules has evolved over the past 20 years from a chemical biology curiosity to a promising therapeutic modality, with clear dose-dependent degradation of therapeutic targets such as AR, IRAK4 or BTK observed in man (clinicaltrials.gov identifiers NCT03888612, NCT04772885, NCT04830137), and the question is no longer whether but when the first PROTAC will be approved for therapeutic use by regulatory agencies. Proximity-induced Pharmacology (ProxPharm) is an extension of targeted protein degradation, where chemically induced proximity with proteins beyond E3 ligases can be used to rewire the molecular circuitry of cells for chemical biology applications or therapeutic benefit [2], [3]. Indeed, ProxPharm compounds were recently reported that recruit a phosphatase, two kinases, an acetyltransferase, and a deubiquitinase to post-translationally modify neosubstrates [4], [5], [6], [7].

Structural studies have shown that PROTACs are not simply acting as chemical linkers but rather stabilize non-natural protein–protein interactions between E3 ligases and target proteins [8]. Because compatible protein interfaces do not always exist between two proteins, a prevailing notion is that a collection of chemical handles binding a diverse array of E3 ligases will be necessary to productively induce the degradation of any given protein. Additionally, the tissue expression profile and subcellular localization of the E3 ligase must match that of the target protein for a PROTAC to be active. Finally, PROTACs recruiting E3 ligases with disease-specific tissue expression profiles can avoid adverse effects associated with the indiscriminate inhibition of the protein target. For example, a senolytic PROTAC exploits the restricted expression profile of the E3 ligase CRBN to avoid toxicity associated with the adverse inhibition of the target protein, Bcl-xl, in platelets [9]. Similar rules are expected to apply to ProxPharm compounds beyond PROTACs, emphasizing the need to identify chemical handles for a diverse array of protein-modifying enzymes.

To uncover novel opportunities for the development of future ProxPharm compounds, we searched for non-catalytic ligandable pockets (structural cavities that can be occupied by small-molecule ligands) in all experimental structures of human protein-modifying enzymes, including kinases, phosphatases, acetyltransferases, deacetylases, methyltransferases, demethylases, glycosyltransferases, glycosidases and deubiquitinases. These pockets need to be non-catalytic to preserve the catalytic activity of the protein-modifying enzyme which is necessary for the ProxPharm-induced response. The ligandability of E3 ligases was previously reviewed and not considered in this analysis which is focused on opportunities for proximity pharmacology beyond PROTACs [1], [10], [11], [12], [13], [14]. We identified non-catalytic pockets in 369 human enzymes, including those recruited by previously reported ProxPharm compounds. This analysis further confirms the rich potential of proximity pharmacology for chemical biology applications.

## 2 Methods

### 2.1 Mapping binding pockets

A list of enzymes was compiled from the Expasy ENZYME database and the UniprotKB database [15] and mapped to corresponding PDB codes. The 3D structures were extracted from the PDB and the biologically relevant oligomeric state was generated with ICM (Molsoft, San Diego). The icmPocketfinder module was run against each converted ICM object using default settings. The pockets were categorized as non-catalytic based on the following two approaches.

### 2.2 Interpro domain analysis

The domain architecture of each enzyme was extracted from the InterPro database [16]. The domains were marked either as catalytic or non-catalytic based on GO ontology or literature. Residues within 2.8 Å of the pocket mesh generated by ICM were considered as lining the pocket, and the N- and C-terminal boundaries of this selection were used to define a ‘pseudo’ sequence for the pocket. These sequences were aligned and compared with the domain architecture of the enzyme to determine the domain location of the pocket. If the pocket was in a manually curated non-catalytic domain, the pocket was marked non-catalytic.

### 2.3 Catalytic residues proximity analysis

For each enzyme, the corresponding catalytic residue information was extracted from either the Mechanism and Catalytic Site Atlas database [17] or UniprotKB database [15]. If the catalytic residues were present in the structure, the distance between the pockets and the catalytic residues were measured. If the pocket was more than 7 Å away from the catalytic residues, it was categorized as non-catalytic.

### 2.4 Additional filters

Nucleotide binding residues and co-factor binding residues information was extracted from the UniprotKB database to determine which pockets corresponded to nucleotide or co-factor binding sites. For example, the ATP binding site in protein kinases or the acetyl-CoA binding site in acetyltransferases. If the distance between the pocket and nucleotide/co-factor binding residues was<7 Å, the pocket was filtered out. If the pocket was in proximity (<5Å) of unresolved residues in the structure due to poor electron density, the pocket was not included for further analysis. If the catalytic residues were among the missing residues, pockets were excluded as well. Pockets were also excluded when located at the interface of inhibitor proteins and enzyme complexes. Next, pockets were filtered for duplicates: when two structures representing the same enzyme had a similar pocket, the largest pocket was retained. Pockets predicted unligandable were also removed: ligandability was determined using the pocket properties generated by ICM (volume: 155.7–661.1 Å3, area: 155–655 Å2, hydrophobicity: >0.44, buriedness: 0.6–0.95, DLID [18]: >-1). Cut-off values were based on properties of experimentally proven druggable pockets. Lastly, the pockets were grouped based on their domains. A list of manually curated non-catalytic domains was formed, from which non-catalytic domains necessary for the catalytic activity were excluded.

### 2.5 Cysteine reactivity

The predicted reactivity of cysteine sidechains lining pockets was predicted using the ReactiveCys module of ICM. The method is based on reactivity data for 34 reactive and 184 non-reactive cysteines from isoTOP-ABPP (isotopic tandem orthogonal proteolysis activity-based protein profiling) [19] and a nonredundant set of PDB protein structures (resolution < 2.5 A) with covalently-modified cysteines (272 reactive).

## 3 Results

**Fig. 1:**
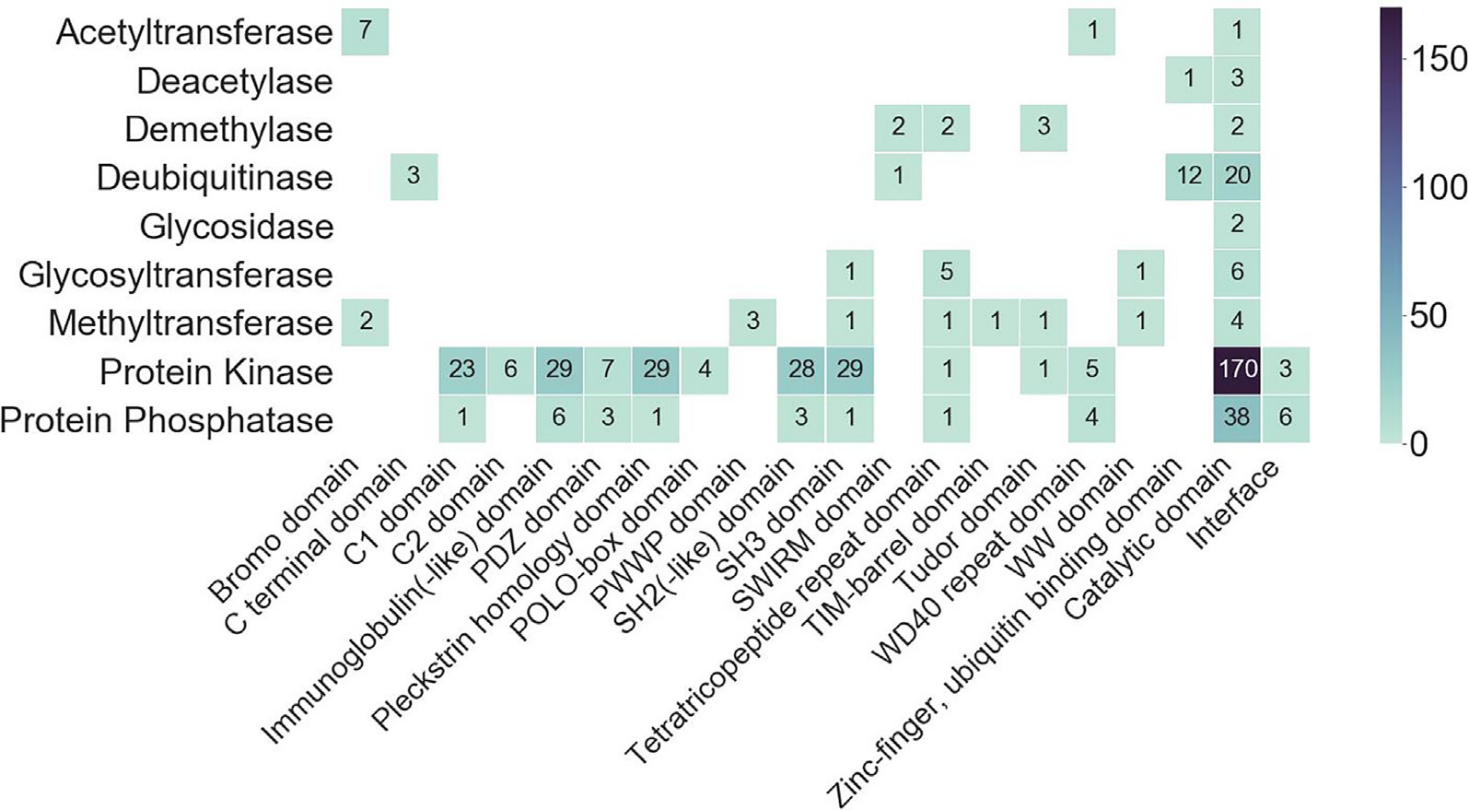
Distribution of non-catalytic pockets in human protein-modifying enzymes. The number of proteins with a putative ligandable non-catalytic pocket is shown for each structural domain and each protein family. For each enzyme family, the total number of proteins in human can be found in Table S1.

To assemble a database of druggable binding pockets that may be exploited by ProxPharm compounds, all high-resolution structures of human protein-modifying enzymes beyond E3 ligases in the PDB were analyzed with the cavity mapping tool IcmPocketFinder (Molsoft, San Diego). Only structural cavities with properties (volume, area, hydrophobicity, buriedness and drug-like density (DLID)) within a pre-defined range (detailed in the Methods section) were deemed ligandable and were considered further. A permissive definition of ligandability was used to reflect the fact that chemical handles for ProxPharm compounds do not need to bind with high potency to their target. Indeed, ligands with up to 10 µM affinity have been successfully used to make PROTACs [20]. When a ligandable cavity was found in a non-catalytic domain, the domain was also deemed ligandable in the context of enzymes not in the PDB, but with a low confidence score. When enzymes were bound to other proteins in the PDB, cavities were also searched at the protein interface. Pockets that may be exploited by ProxPharm compounds could be divided into three categories: 1) those located in non-catalytic domains, 2) those found at non-catalytic sites of the catalytic domain, 3) those mapping at the interface of protein complexes (Fig. 1).

Potentially ligandable non-catalytic pockets were found in 236 kinases, 45 phosphatases, 37 deubiquitinases, as well as several writers and erasers of methyl, acetyl and glycosyl groups (Fig. 1, Table S1-3). In the following section, we review in detail each protein family.

### 3.1 Protein kinases

**Fig. 2:**
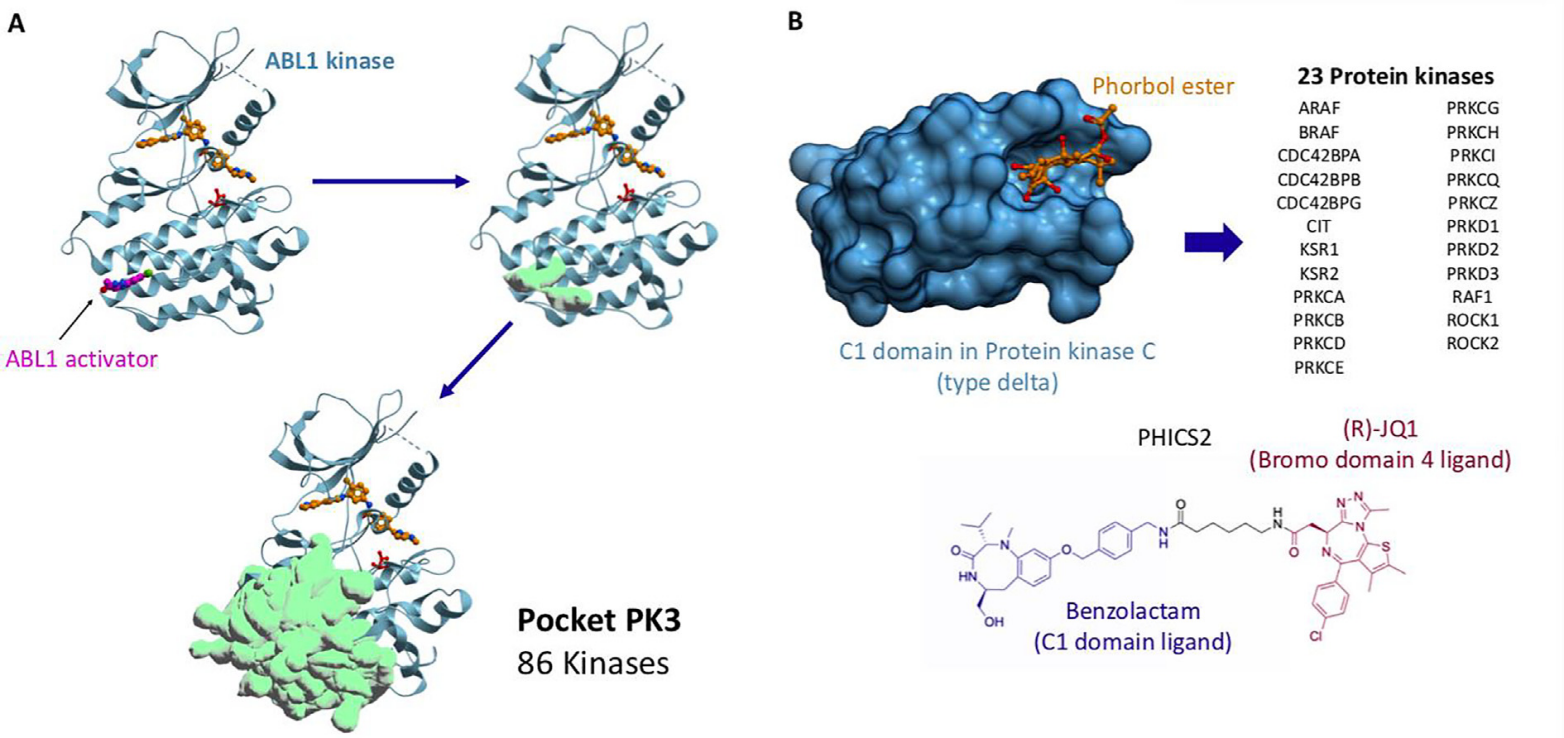
Recurrent non-catalytic pockets found in kinases. A) Pockets found in the kinase domain. ABL1 (blue) bound to catalytic inhibitor (orange) is used as a canonical reference structure (PDB: 6NPU [21]). An allosteric activator is shown in purple. A similar pocket is found in 86 protein kinases. B) Phorbol ester bound to the C1 domain of KPCD (PDB: 1PTR [28]). A C1 domain can be found in 23 protein kinases. Benzolactam binds to C1 domains and is used as chemical handle to recruit protein kinase C (type delta) to Bromodomain-containing protein 4 (PHICS2) [4]. The full list of kinases and pockets summarized here is provided in Table S2, S3 and in the database ProxyBind.

Ligandable non-catalytic pockets were found in the catalytic domain of 170 kinases (Fig. 1, Table S2). For instance, in 86 kinases, a pocket is found in the α-lobe of the kinase domain (Fig. 2A, Pocket PK3) and, in the context of Abelson kinase, is exploited by an activating compound located over 15 Å away from the imatinib-occupied active site (Figure S1, PDB 6NPU, Pocket PK3) [21]. Other pockets are recurrently found at five other locations and could potentially be exploited to pharmacologically hijack kinases (Figure S1). In particular, 47 kinases share a cavity below the sub-activation loop (Figure S1, Pocket PK4) which is occupied by a small molecule in the MAP kinase p38α [22] (PDB 3HVC). A β-lobe cavity is found in another 25 kinases (Figure S1, Pocket PK5), where, in PDK1, a cysteine is covalently engaged by fragment inhibitors or activators (PDB 3ORZ) [23] and a different β-lobe pocket is identified in 9 kinases (Figure S1, Pocket PK2) and occupied by a fragment molecule in the context of CDK2 (PDB 6Q4D) [24].

Ligandable pockets were also found at the interface of the catalytic domain of 3 kinases (PRKAA1, PRKAA2, CDK5) and cofactor proteins (Figure S1). For example, a pharmacological activator is sandwiched at the interface of the β-lobe of PRKAA1 and its cofactor PRKAB1 (Fig. 2B, Pocket PKI1) [25]. Interestingly, this chemical scaffold was recently linked to an inhibitor of Bruton’s tyrosine kinase (BTK), leading to the phosphorylation of BTK by PRKAA1 in cells, in what was the first example of a phosphorylation-inducing chimeric small molecule (PHIC) [4]. Another pocket is found at the interface of CDK5 and CDK5R1, a neural-specific CDK5 activator protein, raising the possibility to develop brain-specific phosphorylating agents that would exploit this site (Figure S1).

Multiple potentially ligandable cavities were also identified in non-catalytic domains of kinases (Table S2, Figure S2). For example, a cavity was found in the non-catalytic C1 domain of 23 kinases such as BRAF, CDC42 binding kinases, or PKC kinases (Fig. 2). Binding of diacylglycerol to this pocket leads to translocation from the cytosol to the membrane of PKC kinases, and catalytic activation [26]. The cavity was successfully targeted by drug-like molecules such as V8-benzolactams [27], which can be used as PKC-recruiting handles in heterobifunctional PHICS. Using this strategy, Siriwardena et al. could induce the phosphorylation of BRD4 by PKC [4]. A similar strategy may be applied to the other kinases where we also identified a C1-domain cavity.

A membrane-targeting C2 domain is also present in 6 protein kinases, including PKC kinases, but the ligandability of its phosphatidylserine binding pocket is unclear. A tyrosine-lined pocket conserved in the POLO domain of PLK kinases participates in substrate recognition and was targeted by weak compounds that would need to be optimized to serve as ProxPharm handles (Figure S2) [29]. Five kinases contain a WD-40 repeat (WDR), which is a β -propeller domain with a druggable central cavity [30]. For instance, the WDR domain of LRRK2 could be exploited by future PHICS to phosphorylate targets in the brain, where it is expressed.

Other protein domains of potential interest were identified in human kinases, but even though cavities meeting our selection criteria were found, the general ligandability of these domains remains to be supported experimentally. For instance, 29 kinases contain an immunoglobulin-like domain (Figure 1 and S2). Small molecule ligands were shown to bind to the immunoglobulin-like domain of the unrelated protein RAGE, but ligands were prohibitively weak [31]. Another 28 kinases contain both SH2 and SH3 domains (Figure 1 and S2), known to participate in the formation of an auto-inhibitory state and contribute to substrate recruitment of Src family kinases. Despite sustained efforts, potent, drug- like, cell-penetrant ligands remain to be found for these domains. Nevertheless, they may be sufficiently ligandable for the discovery of weak compounds that may serve as valid chemical handles for kinase-recruiting ProxPharm molecules. In another example, the poorly characterized kinase STK31 includes a Tudor domain (Fig. 1, Table S2), generally found in proteins involved in chromatin-mediated signaling. This domain was targeted by a potent chemical probe in the context of the methyltransferase SETDB1 [32] and may be ligandable in STK31.

### 3.2 Protein phosphatases

**Fig. 3:**
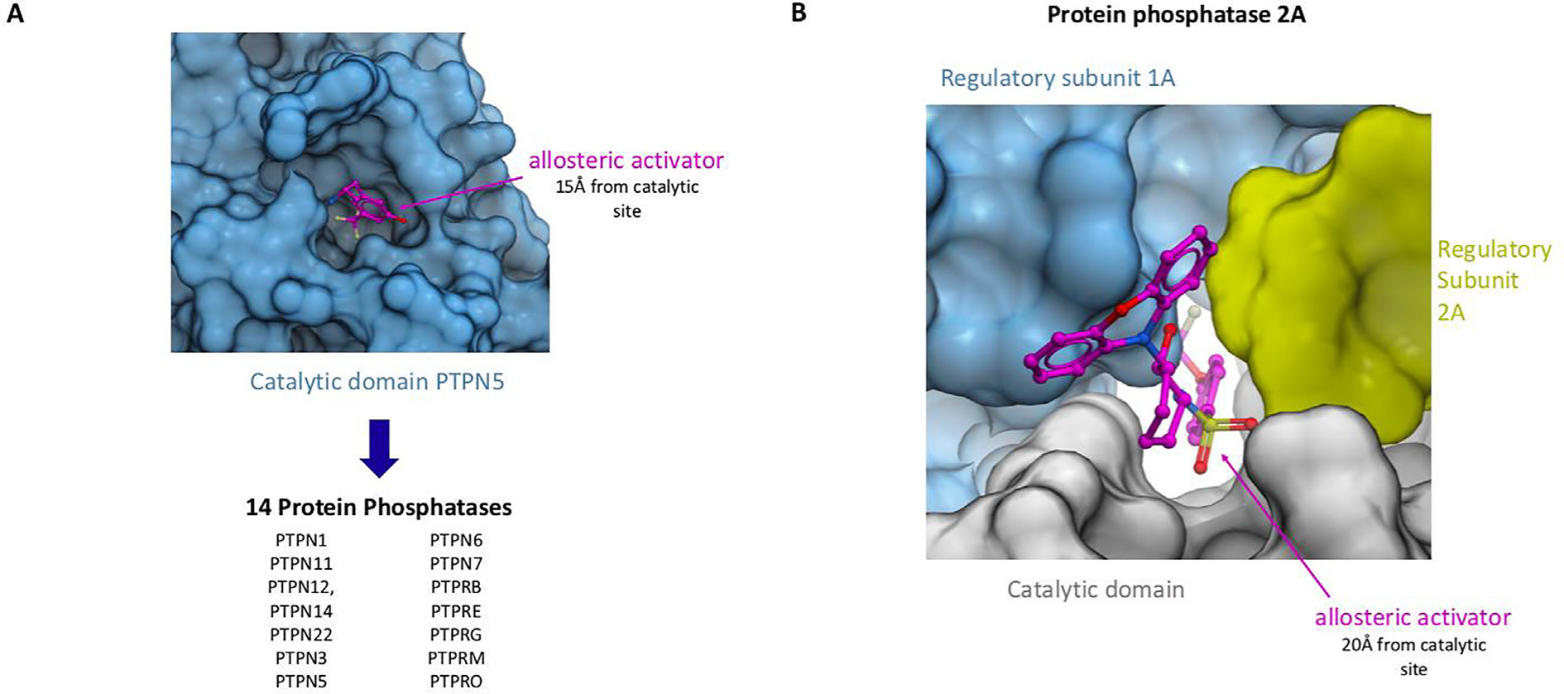
Recurrent non-catalytic pockets mapping at protein phosphatase domains. A) Pocket in the catalytic domain of tyrosine-protein phosphatases 5 bound to an allosteric activator (purple) 15 Å from the catalytic site (PDB 6H8R). A similar pocket (labelled PP3 in Table S3 and the Proxybind database) is recurrent in 14 tyrosine-protein phosphatases. B) Pocket found at the interface of the protein phosphatase 2A catalytic domain (gray) and interacting subunits (blue and yellow) with allosteric activator (PDB: 6NTS) [34].

Non-catalytic pockets were found in 51 protein phosphatases (Table S3). Among these, 38 protein phosphatases have a non-catalytic pocket in the catalytic domain and 20 enzymes in juxtaposed domains (Fig. 1, Table S2). Some of the non-catalytic cavities were recurrently found in the phosphatase domain: 13 tyrosine-protein phosphatases share a cavity 15 Å from the catalytic site (Fig. 3, Pocket PP3), which, in the context of PTPN5, is occupied by an allosteric activator (PDB 6H8R) [33]. The recurrent pocket locations are labelled as pocket Protein Phosphatase # (Pocket PP#). Other recurrent cavities are found at four other locations of the catalytic domain and could potentially be exploited to recruit tyrosine-protein phosphatases to target proteins (Fig. S3A). Furthermore, 5 serine/threonine-protein phosphatases have 4 recurrent non-catalytic cavities in their catalytic domain (Fig. S3B).

Non-catalytic pockets were also found at multiple protein–protein interfaces (locations denoted as Pocket Protein Phosphatases Interface # (Pocket PPI#)), including a cavity located at the interface of the three subunits of the protein phosphatase 2A (PP2A) heterotrimer, and occupied by a small molecule activator [34] (Fig. 3, Pocket PPI1, Figure S4). Heterobifunctional compounds derived from this activator could potentially be used for targeted dephosphorylation. This hypothesis is further supported by the fact that another phosphatase, PP2A, was successfully recruited to dephosphorylate the kinases AKT or EGFR by linking kinase inhibitors to peptidic ligands that exploit the tetratricopeptide repeat domain in PP2A [5].

Cavities are also found in the PDZ domain of protein phosphatases PTPN3, PTPN4 and PTPN14 (Figure S5). The ligandability of these pockets is not experimentally validated, but they are occupied by the C-terminal leucine or valine of pentameric peptide ligands [35], [36], and a similar pocket in the PDZ domain of the unrelated protein PICK1 was crystallized in complex with a small molecule binding with sub-micromolar potency [37]. Finally, pockets with unclear ligandability were found in the SH2 domain of phosphatases PTPN6, PTPN11 and TNS2, and the tetratricopeptide repeat of PPP5C (Figure S5).

### 3.3 Protein methyltransferases

**Fig. 4:**
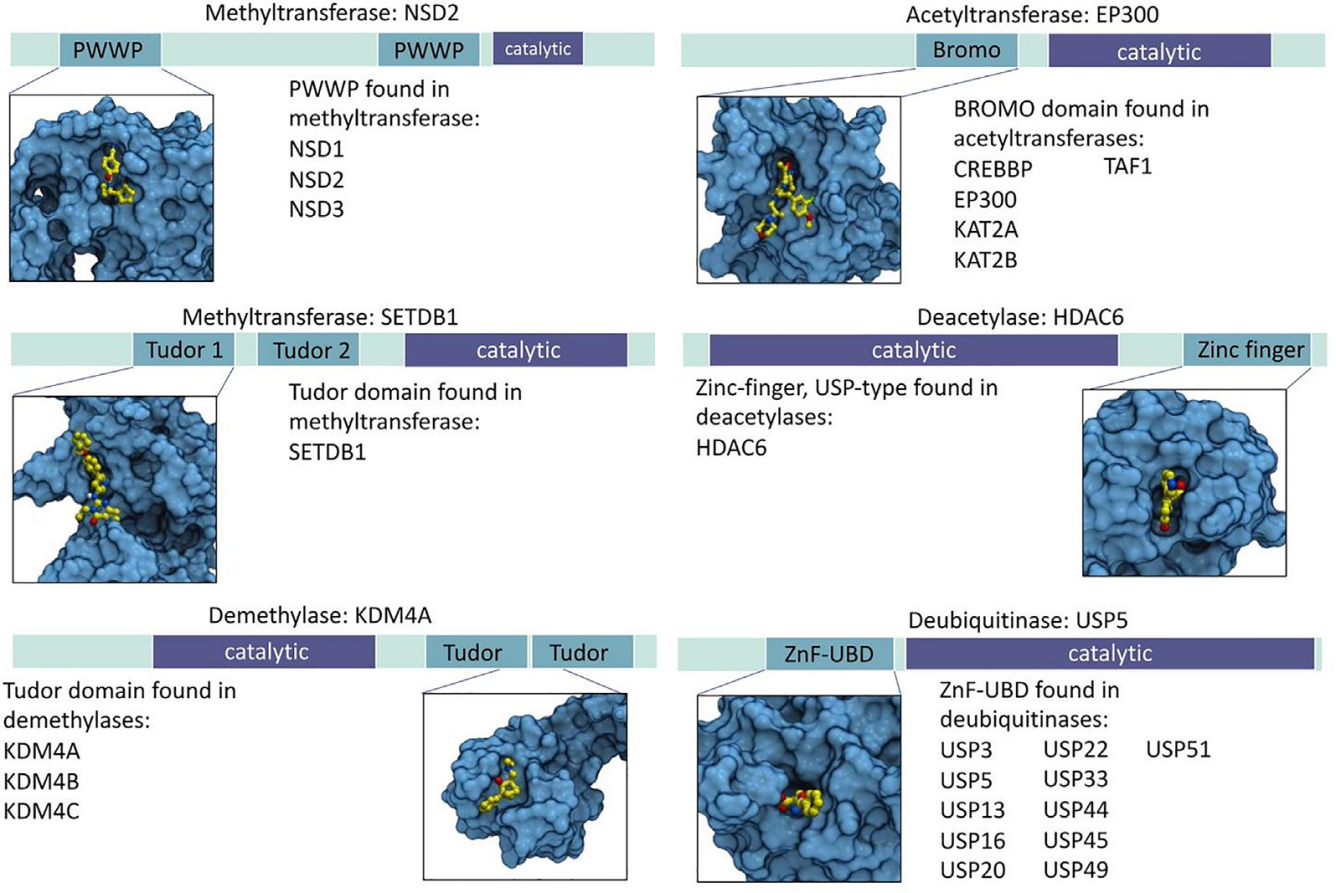
Examples of pockets in non-catalytic domains of methyltransferases, acetyltransferases, demethylases, deacetylases and deubiquitinases. PWWP domain in NSD2 with small-molecule ligand (PDB: 6UE6 [55]), bromodomain in EP300 with small-molecule ligand (PDB: 5BT3), Tudor domain in SETDB1 with small-molecule ligand (PDB: 7CJT [32]), zinc-finger, UBP-type in HDAC6 with small-molecule ligand (PDB: 5KH7 [56]), Tudor domain in KDM4A with chemical fragment (PDB: 5VAR [46]), ZnF-UBD in USP5 with small-molecule ligand (PDB: 6DXT [51]).

Protein methyltransferases (PMTs) are typically large multi-modular proteins where chromatin-binding binding modules are often found juxtaposed to the catalytic domain. For instance, a PWWP domain is found in the NSD subfamily of PMTs (NSD1, NSD2 and NSD3) and chemical probes were reported for the PWWP domain of NSD2 and NSD3 (Fig. 4) [38], [39]. The NSD3 ligand was recently used as the chemical handle of an NSD3-degrading PROTAC [40]. These ligands - which do not inhibit the enzymatic activity – could also potentially serve as chemical moieties to recruit NSD2 or NSD3 for the methylation of new protein substrates.

SETDB1, another multi-modular PMT, includes a non-catalytic Tudor domain selectively targeted by a potent chemical probe that may be linked to other ligands to methylate non-natural protein substrates (Fig. 4) [32]. Interestingly, recurrent genetic aberrations drive the overexpression of NSD2 in multiple myeloma and pediatric leukemia, and of NSD2, NSD3 and SETDB1 in lung cancer [41], [42], [43], [44], which could possibly offer an opportunity for targeted protein methylation in cells presenting a specific disease-associated genetic profile. Putative ligandable cavities were found in a few other non-catalytic domains of PMTs, including the bromodomain of KMT2A and ASH1L (bromodomains are typically druggable (Fig. 1) [45], but no ligand was reported for these domains.

A recurrent pocket was also found in the catalytic domain of two protein arginine methyltransferases, PRMT3 and PRMT8, which is located more than 17 Å away from the catalytic site (Figure S6, Pocket M1). Other unique non-catalytic pockets were found in the methyltransferase catalytic domain of 3 PMTs (PRMT3, SETD7, CARM1) (Table S3). These cavities met our ligandability criteria but so far, their chemical tractability was not validated experimentally.

### 3.4 Lysine demethylases

A number of non-catalytic domains of lysine demethylases include potentially ligandable pockets. KDM4A, KDM4B and KDM4C all have a Tudor domain, which was shown to be chemically tractable in the context of SETDB1. The Tudor domain of KDM4A was crystallized in complex with a low-affinity chemical fragment (K_D_ ∼ 80 µM) that may be optimized into a stronger-binding chemical handle towards the development of a demethylase-recruiting bifunctional molecules (Fig. 4) [46]. Putative ligandable pockets were also found in the tetratricopeptide repeat of KDM6A and UTY and the SWIRM domain of KDM1A and KDM1B (Fig. 1, Table S2), but no ligand was so far reported for these domains.

### 3.5 Lysine acetyltransferases

With over 3000 acetylated lysine sidechains across 1700 human proteins, acetylation is a ubiquitous post-translational modification involved in a diverse array of cellular machineries such as the regulation of gene expression, splicing or cell cycle [47], [48]. Out of 35 lysine acetyltransferases in the human genome, we found non-catalytic ligandable pockets in 9 (Fig. 1, Table S3). Several acetyltransferases include an acetyl-lysine binding bromodomain, five of which were crystallized in complex with multiple small-molecule ligands (EP300, CREBBP, KAT2A, KAT2B and TAF1) (Fig. 4) [45]. A compound targeting the bromodomain of one of these, EP300, was chemically linked to an FKBP12-binding molecule to successfully induce the acetylation of FKBP12-fusion proteins by EP300, thereby confirming that acetyltransferases are amenable to proximity pharmacology, and strongly suggesting that bromodomain ligands could be used as chemical handles to recruit other acetyltransferases to neosubstrates [7].

A WDR domain is also found in GTF3C4, a poorly characterized acetyltransferase (Fig. 1, Table S2). The structure of this domain was not experimentally solved, but WDR domains are ligandable in the context of other proteins [30], [49] and this enzyme could potentially be harnessed for targeted acetylation.

### 3.6 Lysine deacetylases

Deacetylases have a limited number of non-catalytic domains and a ligandable site was found in only one of them: the zinc-finger ubiquitin-binding domain (Znf-UBD) of HDAC6 (Fig. 4). This binding pocket recognizes the C-terminal extremity of ubiquitin and was successfully targeted by small molecule ligands [50] representing excellent chemical handles for proximity pharmacology applications. Non-catalytic pockets were also found in the catalytic domain of three other deacetylases: HDAC4, HDAC8 and HDAC1, but the ligandability of these sites remains to be experimentally validated (Figure S7).

### 3.7 Deubiquitinases

Deubiquitinases (DUBs) typically remove ubiquitin tags deposited by E3 ligases. When these tags are signalling for proteasomal degradation, DUBs deubiquitinate and rescue their protein substrates from the ubiquitin–proteasome system and have a stabilizing effect on their target. Chemical handles binding non-catalytic pockets of DUBs may therefore enable the recruitment of DUBs for targeted protein stabilization. As a proof-of-concept, a bifunctional molecule linking a ligand that covalently engages the DUB OTUB1 to a chemical moiety that binds ΔF508-CFTR in cystic fibrosis could stabilize ΔF508-CFTR in an OTUB1-dependent manner [6]. There is no structural information on the *N*-terminal domain of OTUB1 that is covalently recruited by this chimeric compound, but structures of other non-catalytic domains in DUBs reveal other opportunities for targeted protein stabilization.

The most recurrent ligandable non-catalytic domain of DUBs is the Znf-UBD, found in 12 ubiquitin-specific proteases (USPs, a class of DUBs) (Figure S7, Table S2). Low micromolar ligands were reported for the Znf-UBD of USP5, but these compounds were shown to inhibit the catalytic activity of USP5 and therefore cannot be used as chemical handles to productively recruit USP5 to neosubstrates [51]. However, the function of the Znf-UBD of DUBs is poorly understood in other USPs, and ligands targeting this domain may still be valid handles for targeted protein stabilization in the context of other DUBs.

Ligandable pockets were also found in a tandem ubiquitin-like domain located at the C-terminus of four DUBs: USP7, 11, 15 and 25 (Fig. 1, Table S2). In the context of USP7, this domain binds and activates the catalytic domain [52]. In the absence of structure of full-length USP7 in its activated form, it is unclear whether ligands occupying this C-terminal binding pocket would preserve the activation mechanism of USP7 and could be used to productively recruit USP7 for targeted protein stabilization. Another non-catalytic domain present in deubiquitinases is a SWIRM domain in MYSM1. Chemical ligands have not yet been reported for this domain. Non-catalytic pockets were recurring at six locations of eight USPs within the peptidase C19-type catalytic domain (Figure S8A, Table S3). Another non-catalytic cavity is observed in the peptidase C12-type catalytic domain of UCHL1 and UCHL5 (Figure S8B, Table S3). As above, the ligandability of these pockets needs to be confirmed experimentally.

### 3.8 Glycosyltransferases

Glycosylation is a post-translational modification that is most common in excreted and extracellular membrane-associated proteins and is frequently dysregulated in diseases, such as cancer or bacterial infection [53]. Proof of principle for proximity-induced glycosylation of target proteins was established by fusing substrate-targeting nanobodies to the glycosyltransferase O-GlcNActransferase (OGT), which effectively induced the glycosylation of the desired protein targets [54]. Putative ligandable pockets in the tetratricopeptide repeat of OGT and TMTC1-4 may be exploited to chemically recruit these glycosyltransferases to neosubstrates. Similarly, the SH3 domain of FUT8 and WW domain of GALNT9 may be considered for the chemical recruitment of these enzymes. Non-catalytic cavities in the glycosyltransferase domain of ST8SIA3, B3GAT1-3, and POFUT2 were also found but, as above, their ligandability should be confirmed experimentally.

### 3.9 Glycosidases

Similar to glycosyltransferases, protein constructs have been developed using O-GlcNAcase or sialidase connected to nanobody to artificially induce deglycosylation [57], [58], [59]. There are limited structures and domain information available for glycosidases, but ligandable pockets are found in the catalytic domain of OGA and MAN1B1 that could be explored for deglycosylation-inducing chimeras.

### 3.10 Reactive cysteines

PROTACs covalently engaging an E3-ligase have demonstrated that covalent binding is a valid strategy for proximity-induced post-translational modification of target proteins [60], [61], [62], [63], [64]. For instance, covalent recruitment of only a small fraction of the cellular pool of the E3-ligase DCAF16 is sufficient to support targeted degradation [61]. A deubiquitinase-targeting chimera also forms a covalent bond with a cysteine of the DUB OTUB1 [6]. Electrophilic chemical handles enable the covalent recruitment of domains otherwise not considered ligandable, such as the RING domain of the E3-ligase RNF4 [60], and can be advantageous to enhance potency or selectivity. We used ICM to evaluate the reactivity of cysteine sidechains found in non-catalytic pockets of human protein-modifying enzymes (see Methods section for details).

Reactive cysteines were predicted in multiple proteins (Figure S9, Table S3). For instance, C576 is lining a pocket in the UBL domain of USP7 C-terminal to the catalytic domain, C210 is found at an ectopic site of the STK16 kinase domain, C266 at a non-catalytic site of the PP2BA phosphatase domain, and C1030 at a cavity remote from the active site of the deacetylase HDAC4 (Figure S9). It would be interesting to screen such proteins with electrophilic fragments to find covalent adducts that may serve as a starting point for novel proximity-pharmacology applications.

## 4 Discussion

Our systematic structural survey of the human proteome reveals numerous opportunities for the pharmacological recruitment of protein-modifying enzymes beyond E3 ligases to non-natural substrates. The predicted ligandability of a binding pocket can vary from one method to another and is not a conclusive metric. Here, we use a permissive definition based on volume, area, hydrophobicity, buriedness and DLID values. We first note that, in addition to the hundreds of new pockets identified, this approach does retrieve binding sites for known ProxPharm compounds, including a protein–protein interface pocket used to recruit the kinase PRKAA (Fig. S1B, Pocket PKI1) [4] and a bromodomain pocket used to recruit the acetyltransferase EP300 (Fig. 4) [7].

Among the collection of binding sites that we compiled, we assigned the highest confidence (confidence level 1, Table S3-4) to the ones for which a high-affinity ligand was already reported. Table with ligandability confidence scale can be found in the Supplementary information (Table S4). For instance, V8-benzolactams bind the C1 domain of protein kinase C (Fig. 2B) [4], [27], UNC6934 binds the PWWP domain of NSD2 (Fig. 4) [39] and compound R734 binds a protein interface of the kinase AMPK (Fig. S1B) [4], [25]. A number of non-catalytic pockets were also found that are targeted by weak ligands that may be valid starting points for the development of ProxPharm compounds (confidence level 2, Table S3-4). These include compounds and peptides found in the POLO-box domain of PLK1 (Figure S2) [29] and the PDZ domain of PTPN3 (Figure S5) [35]. Because ProxPharm compounds induce the formation of ternary complexes where direct protein–protein interactions contribute to the overall energy of the system, proximity-inducing compounds can be derived from chemical handles binding with relatively weak affinity (up to 10 µM or more) to their target (Han et al. 2019). Binding sites with this lower confidence level therefore remain of potential interest. Less reliable, but still promising are domains for which no ligand was reported in the context of the protein of interest, but that were shown to be chemically tractable in other proteins (confidence level 3, Table S3-4). For example, low nanomolar ligands targeting the WDR domains of EED and WDR5 are in pre-clinical [65], [66], [67] or clinical development (EED clinicaltrials.gov identifier NCT02900651) and WDR domains are found in the kinases LRRK1, LRRK2, MET, MST1R, PIK3R4 and the acetyltransferase GTF3C4 (Table S2). Similarly, Tudor domains are found in demethylases (KDM4A, KDM4B, KDM4C) and protein kinase STK31 (Table S2), and share a canonical aromatic cage with the Tudor domain of SETDB1 targeted by a high-affinity ligand (K_D_ 90 nM) [32]. Finally, sites that meet our ligandability criteria but for which no ligands were found in the protein of interest or close homologues are less reliable (confidence level 4, Table S3-4).

A limitation of our analysis is that we focused exclusively on the structures of enzymes that add or remove chemical or peptidic tags to proteins. In the future, we believe it would be interesting to expand to other enzymes, such as proteases. Pockets in non-catalytic protein subunits of enzymatic complexes may also serve as starting points for ProxPharm development. Lastly, proteins without enzymatic activity, for example transcription factors, could be analyzed for ligandable pockets that could be recruited with ProxPharm compounds. While beyond the scope of this work, such studies could be undertaken using methodologies similar to the ones presented here. We also limited our approach to proteins (and homologs) with structural information in the protein databank, but recent breakthroughs in protein structure predictions [68], [69], [70] may enable a future expansion of the analysis to the entire human proteome. Finally, the design of hetero-bifunctional molecules with favourable ADME profiles able to productively induce protein–protein interactions in a structural arrangement that allows enzymatic activity of one protein onto the other remains a challenging trial-and-error process. The necessary combinatorial synthesis of candidate molecules with varying chemical handles and linkers, and subsequent screening are non-trivial experimentally. Recent signs of progress in the rational design of PROTACs should nevertheless be noted [71], [72], [73], [74], [75].

In spite of these limitations, the compendium of binding sites – including some with chemical starting points - for proximity-induced pharmacology assembled here reveals a multitude of avenues to harness protein modifying enzymes involved in epigenetic mechanisms, splicing, protein homeostasis and other cellular machineries. For example, recruiting histone methyltransferases or acetyltransferases to specific genomic loci may up-regulate repressed genes, while targeted glycosylation may be a mechanism to control inflammatory and viral immune responses. Now that molecular proof-of-concept was established for a number of enzyme classes, it is the time to fully explore and test the boundaries of this promising modality for chemical biology and drug discovery applications.

## CRediT authorship contribution statement

**Evianne Rovers:** Data curation, Formal analysis, Investigation, Methodology, Visualization, Writing – original draft, Writing – review & editing. **Lihua Liu:** Data curation, Visualization. **Matthieu Schapira:** Conceptualization, Formal analysis, Funding acquisition, Investigation, Methodology, Writing – review & editing.

## Declaration of Competing Interest

The authors declare that they have no known competing financial interests or personal relationships that could have appeared to influence the work reported in this paper.
