# Supplementary material for: ProxyBind: A compendium of binding sites for proximity-induced pharmacology
Source: Comput Struct Biotechnol J. 2022 Nov 8;20:6163–71. doi: 10.1016/j.csbj.2022.11.010 (PMC9674861; doi:10.1016/j.csbj.2022.11.010)
Supplement: Supplementary data 1 [file mmc1.docx]

**ProxyBind: a Compendium of Binding Sites for Proximity-Induced Pharmacology**

Evianne Rovers^1^, Lihua Liu^1^, Matthieu Schapira^1,2,*^

^1^Structural Genomics Consortium, University of Toronto, Toronto, ON M5G 1L7, Canada,

^2^Department of Pharmacology and Toxicology, University of Toronto, Toronto, ON M5S 1A8, Canada

*To whom correspondence should be addressed.

Contact: [matthieu.schapira@utoronto.ca](mailto:matthieu.schapira@utoronto.ca)

**Appendix A. Supporting information**

Table S1. Distribution of non-catalytic pockets in human protein-modifying enzymes.............p. 3

Table S2: Putative ligandable domains (defined by their Interpro ID) and associated list of human protein-modifying enzymes......................................................................................p. S-4-5

Table S3: Ligandable non-catalytic pockets in protein modifying enzymes including acetyltransferases, deacetylases, methyltransferases, demethylases, glycosyltransferases, glycosidases, deubiquitinases, protein kinases and protein phosphatases................................p. S-6

Table S4: Ligandability Confidence Rating Scale...................................................................p. S-7

Figure S1. Recurrent non-catalytic pockets mapping at protein kinase domains....................p. S-8

Figure S2: Examples of non-catalytic pockets found in diverse domains of protein kinases..p. S-9

Figure S3: Recurrent non-catalytic pockets in catalytic domain of protein phosphatases.....p. S-10

Figure S4: Pockets found at the interface of the protein phosphatase domain and interacting protein domains......................................................................................................................p. S-11

Figure S5: Examples of non-catalytic pockets in protein phosphatases................................p. S-12

Figure S6: Recurrent non-catalytic pockets in catalytic domain of Protein arginine methyltransferases..................................................................................................................p. S-13

Figure S7: Recurrent non-catalytic pockets in the catalytic domain of histone deacetylases............................................................................................................................p. S-14

Figure S8: Recurrent non-catalytic pockets in the catalytic domain of peptidases................p. S-15

Figure S9: Examples of non-catalytic pockets with reactive cysteine residue lining the cavity......................................................................................................................................p. S-16

References:........................................................................................................................p. S-17-19

Table S1. Distribution of non-catalytic pockets in human protein-modifying enzymes. The number of proteins with a putative ligandable non-catalytic pocket is shown for each protein family.

| Protein Family | Number of protein-modifying enzymes with non-catalytic pocket | Total number of enzymes in protein family |
| --- | --- | --- |
| Acetyltransferase | 11 | 35 [1] |
| Deacetylase | 4 | 18 [1] |
| Demethylase | 7 | 27 [1] |
| Deubiquitinase | 37 | 114 [2] |
| Glycosidase | 2 | 10 [3] |
| Glycosyltransferase | 13 | 168 [3] |
| Methyltransferase | 14 | 70 [1] |
| Protein Kinase | 236 | 482 [4] |
| Protein Phosphatase | 45 | 129 [5] |

**Table S2: Putative ligandable domains (defined by their Interpro ID**[6]**) and associated list of human protein-modifying enzymes**

Acetyltransferases, Deacetylase, Demethylase, Deubiquitinase, Glycosidase, Glycosyltransferase, Methyltransferase, Protein Kinase, Protein Phosphatase

| **Domain Name** | **InterPro ID** | **Protein names** |
| --- | --- | --- |
| Bromo domain | IPR001487, IPR018359 | ASH1L, BRD4, CREBBP, EP300, KAT2A, KAT2B, KMT2A, TAF1, TAF1L |
| C1 domain | IPR002219, IPR002420 | ARAF, BRAF, CDC42BPA, CDC42BPB, CDC42BPG, CIT, KSR1, KSR2, PRKCA, PRKCB, PRKCD, PRKCE, PRKCG, PRKCH, PRKCI, PRKCQ, PRKCZ, PRKD1, PRKD2, PRKD3, RAF1, ROCK1, ROCK2, TNS2 |
| C2 domain | IPR002420, IPR000008 | PKN2, PRKCA, PRKCB, PRKCE, PRKCG, PRKCH |
| Immunoglobulin(-like) domain | IPR007110, IPR013783, IPR003599, IPR003598 | ALPK2, ALPK3, AXL, CSF1R, FGFR1, FGFR2, FGFR3, FGFR4, FLT1, FLT3, FLT4, KALRN, KDR, KIT, MERTK, MUSK, MYLK, NTRK1, NTRK2, NTRK3, OBSCN, PDGFRA, PDGFRB, PTK7, PTPRD, PTPRF, PTPRK, PTPRM, PTPRS, PTPRT, ROR1, ROR2, SPEG, TEK, TRIO, TTN, TYRO3 |
| PDZ domain | IPR041489, IPR001478 | CASK, LIMK1, LIMK2, MAST1, MAST2, MAST3, MAST4, PTPN13, PTPN3, PTPN4 |
| POLO box domain | IPR000959, IPR033695 | PLK1, PLK2, PLK3, PLK4 |
| PWWP domain | IPR000313 | NSD1, NSD2, NSD3 |
| Pleckstrin homology domain | IPR001849, IPR041381,  IPR043969, IPR033931 | AKT1, AKT2, AKT3, BMX, BTK, CDC42BPA, CDC42BPB, CDC42BPG, CIT, GRK2, GRK3, ITK, JAK1, JAK2, JAK3, KALRN, MAP3K15, MAP3K5, MAP3K6, OBSCN, PDPK1, PHLPP1, PRKD1, PRKD2, PRKD3, ROCK1, ROCK2, TEC, TRIO, TYK2 |
| SH2(-like) domain | IPR000980 | ABL1, ABL2, BLK, BMX, BTK, CSK, FER, FES, FGR, FRK, FYN, HCK, ITK, JAK1, JAK2, JAK3, LCK, LYN, MATK, PTK6, PTPN11, PTPN6, SRC, SRMS, SYK, TEC, TNS2, TXK, TYK2, YES1, ZAP70 |
| SH3 domain | IPR001452, IPR035750 | ABL1, ABL2, BLK, BTK, CASK, CSK, FGR, FRK, FUT8, FYN, HCK, ITK, KALRN, LCK, LYN, MAP3K10, MAP3K11, MAP3K21, MAP3K9, MATK, OBSCN, PRMT2, PTK6, SRC, SRMS, TEC, TNK1, TNK2, TRIO, TXK, UBASH3B, YES1 |
| SWIRM domain | IPR007526 | KDM1A, KDM1B, MYSM1 |
| TIM barrel domain | IPR035247 | PRMT5 |
| Tetratricopeptide repeat | IPR001440, IPR019734,  IPR013026, IPR006597 | EEF2K, KDM6A, OGT, PPP5C, PRMT9, TMTC1, TMTC2, TMTC3, TMTC4, UTY |
| Tudor domain | IPR041292, IPR002999 | KDM4A, KDM4B, KDM4C, SETDB1, STK31 |
| Ubiquitin carboxyl-terminal hydrolase, C-terminal | IPR029346 | USP7, USP11, USP15 |
| WD 40 repeat | IPR001680, IPR018391,  IPR018391, IPR019775,  IPR017986, IPR036322,  IPR015943 | GTF3C4, LRRK1, LRRK2, MET, MST1R, PIK3R4, ﻿PPP2R2A, PPP2R2B, PPP2R2C, PPP2R2D |
| WW domain | IPR001202 | GALNT9, SETD2 |
| Zinc finger, UBP-type | IPR001607 | HDAC6, USP3, USP5, USP13, USP16, USP20, USP22, USP33, USP39, USP44, USP45, USP49, USP51 |

**Table S3: Ligandable non-catalytic pockets in protein modifying enzymes including acetyltransferases, deacetylases, methyltransferases, demethylases, glycosyltransferases, glycosidases, deubiquitinases, protein kinases and protein phosphatases**

Dataset containing all the identified pockets and pocket characteristics. (The database in .csv format; see Supplementary_table_S3.csv)

**Table S4: Ligandability Confidence Rating Scale**

| **Confidence level** | **Criteria** |
| --- | --- |
| 1 | Non-catalytic pocket for which a high-affinity ligand is reported |
| 2 | Non-catalytic pocket for which a low-affinity ligand, fragment and/or peptide is reported |
| 3 | Non-catalytic pocket for which a ligand, fragment or peptide is reported in a homologous protein or domain |
| 4 | Non-catalytic pocket is predicted ligandable but no ligands were found in the PDB for the protein of interest or close homologues. |


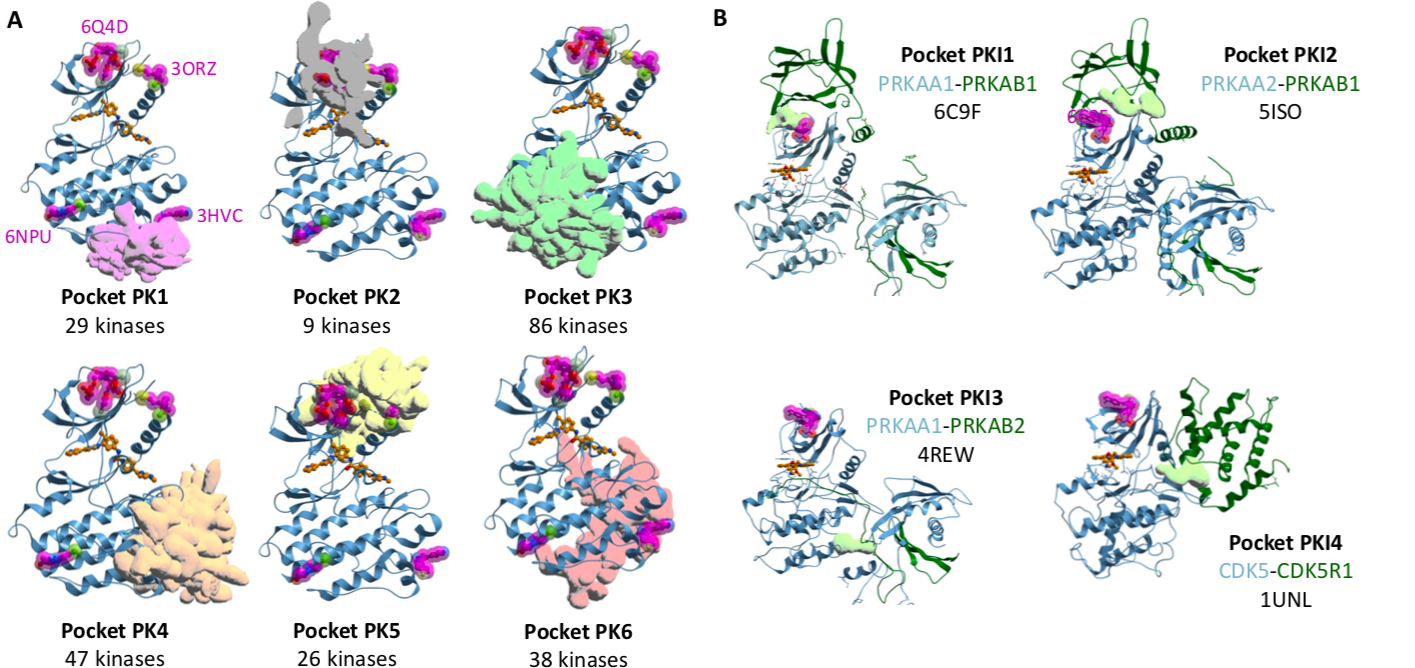


**Figure S1. Recurrent non-catalytic pockets mapping at protein kinase domains.** A) Pockets found in the kinase domain. ABL1 (blue) bound to catalytic inhibitor (orange) is used as a canonical reference structure (PDB 6NPU [7]). Recurrent pockets are shown as overlapping meshes colored based on their location. Allosteric ligands are shown in purple as references on all structures (PDB 6Q4D [8], 3ORZ [9], 6NPU [7], 3HVC [10]). B) Pockets (light green) found at the interface of the kinase domain (blue) and interacting proteins (dark green) in complex structures of PRKAA1 (PDB 6C9F [11], 4REW [12]), PRKAA2 (PDB 5ISO), and CDK5 (PDB 1UNL [8]). Catalytic and allosteric ligands are shown in orange and purple as a reference on all structures (reference ligands PDB 6C9F [6]). The full list of kinases and pockets summarized here is provided in in the database ProxyBind and Table S2.


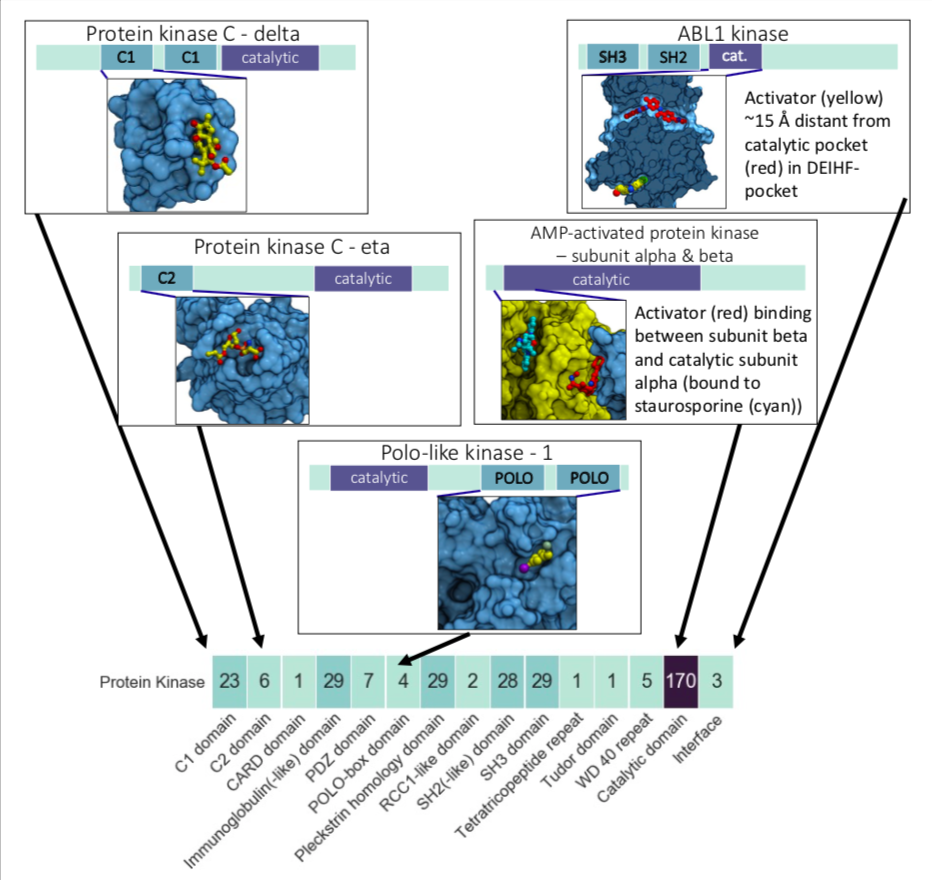


**Figure S2. Examples of non-catalytic pockets found in diverse domains of protein kinases.** Diacylglycerol bound to the C1 domain of KPCD (PDB 1PTR [9]), phosphatidylserine (PDB 1DSY [10]) bound to the C2 domain of PRKCH (PDB 4NDL [11]), fragment bound to the POLO domain of PLK1 (PDB 5NJE [12]), allosteric activators bound to the catalytic domain of ABL1 (PDB 6NPV [2]) and PRKAA (PDB 6C9F [6]). Domain frequency in enzymes is displayed in Table S1. The full list of kinases and pockets summarized here is provided in in the database ProxyBind and Table S2.


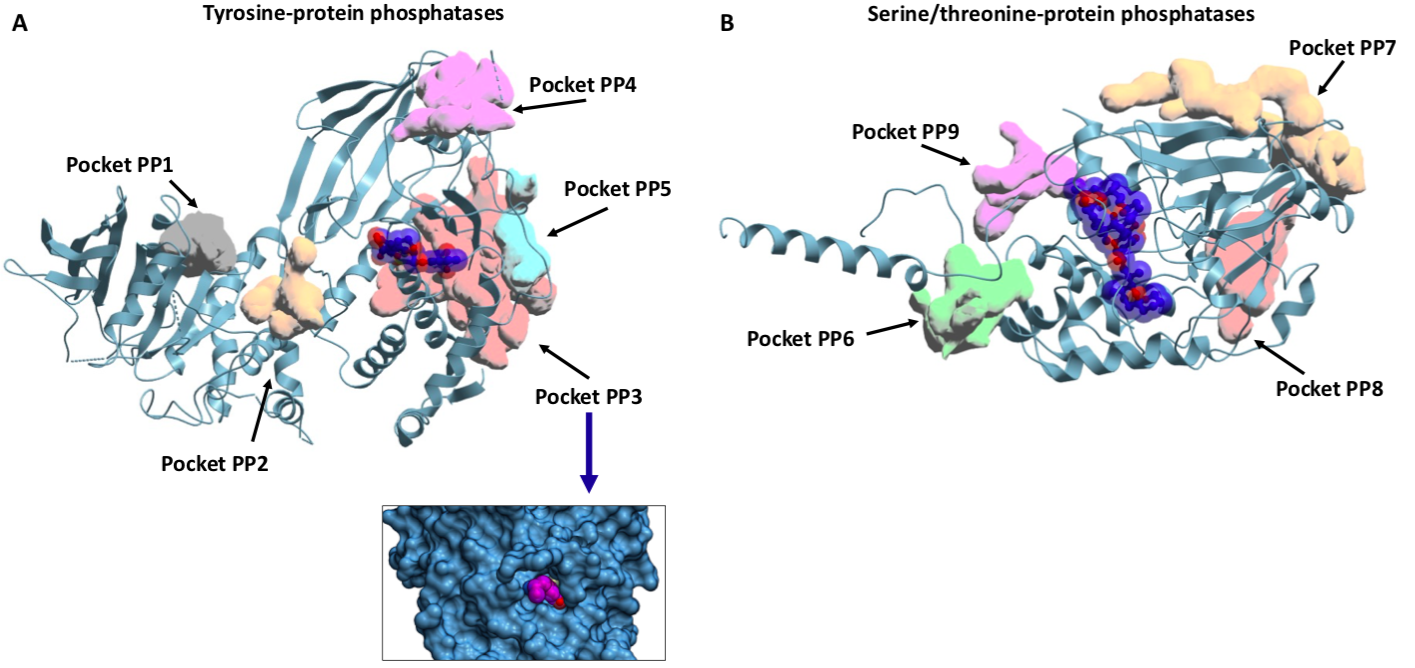


**Figure S3. Recurrent non-catalytic pockets in catalytic domain of protein phosphatases.** A) Tyrosine-protein phosphatases. Reference structure: PDB 2NLK; reference catalytic inhibitor (dark blue): PDB 1L8G [13]; reference allosteric ligand in pocket PP3: PDB 6H8R (purple), B) Serine/threonine-protein phosphatases. Reference structure: PDB 1AUI [14]; reference catalytic inhibitor: PDB 2IE4 (dark blue)[20]. The full list of kinases and pockets summarized here is provided in in the database ProxyBind and Table S2.


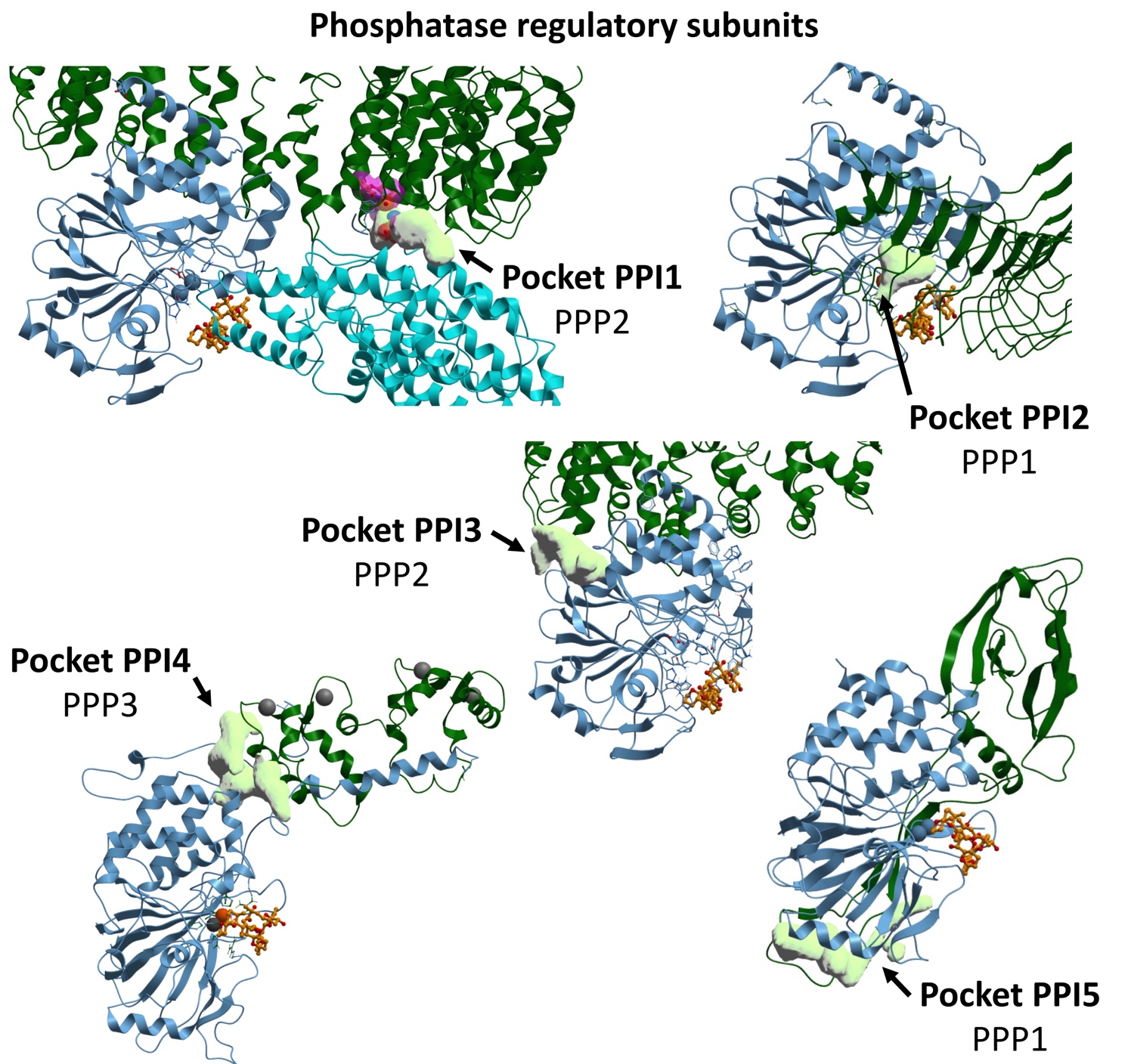


**Figure S4. Pockets found at the interface of the protein phosphatase domain (blue) and interacting protein domains (cyan and dark green).** A catalytic ligand is shown in orange as a reference on all structures (reference ligand PDB 3K7V [16]). Pockets are depicted as green mesh and allosteric activator (PDB 6NTS [17]) is shown in purple. The full list of phosphatases and pockets summarized here is provided in in the database ProxyBind and Table S2.


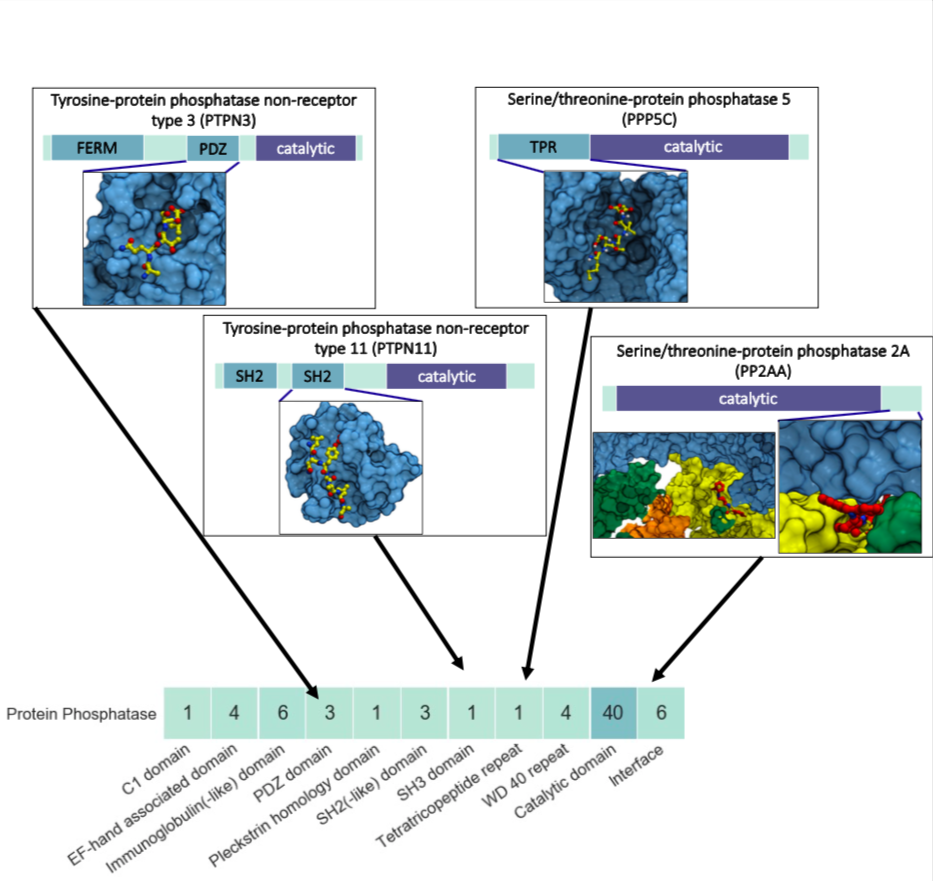


**Figure S5. Examples of non-catalytic pockets in protein phosphatases.** Pockets in PDZ domain (PDB 6HKS [18]), SH2 domain (PDB 6CMP [19]) and tetratricopeptide repeat with bound peptide (PDB 1ELR [20] and 2L6J [20]). Allosteric activator for PP2AA (PDB 6NTS [17]) that binds to an allosteric pocket on the interface of the catalytic domain and regulatory proteins. Domain frequency in enzymes is displayed in Table S1. The full list of kinases and pockets summarized here is provided in in the database ProxyBind and Table S2.


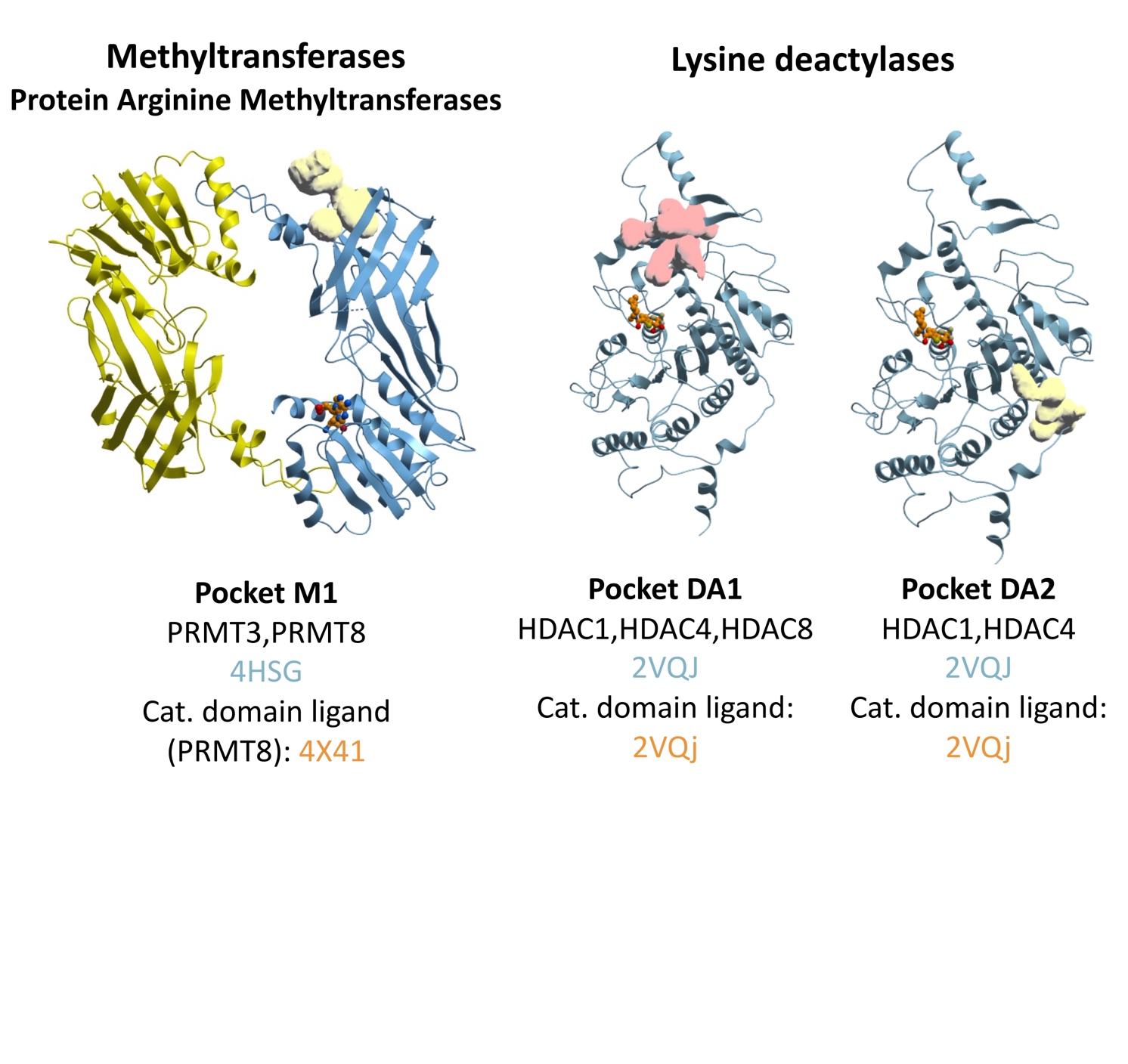


**Figure S6. Recurrent non-catalytic pockets in catalytic domain of Protein arginine methyltransferases.** Protein Arginine methyltransferase (blue) bound to catalytic inhibitor (orange). Reference structure: PDB 4HSG [21]; reference catalytic inhibitor (orange). The full list of methyltransferases and pockets summarized here is provided in in the database ProxyBind and Table S2.


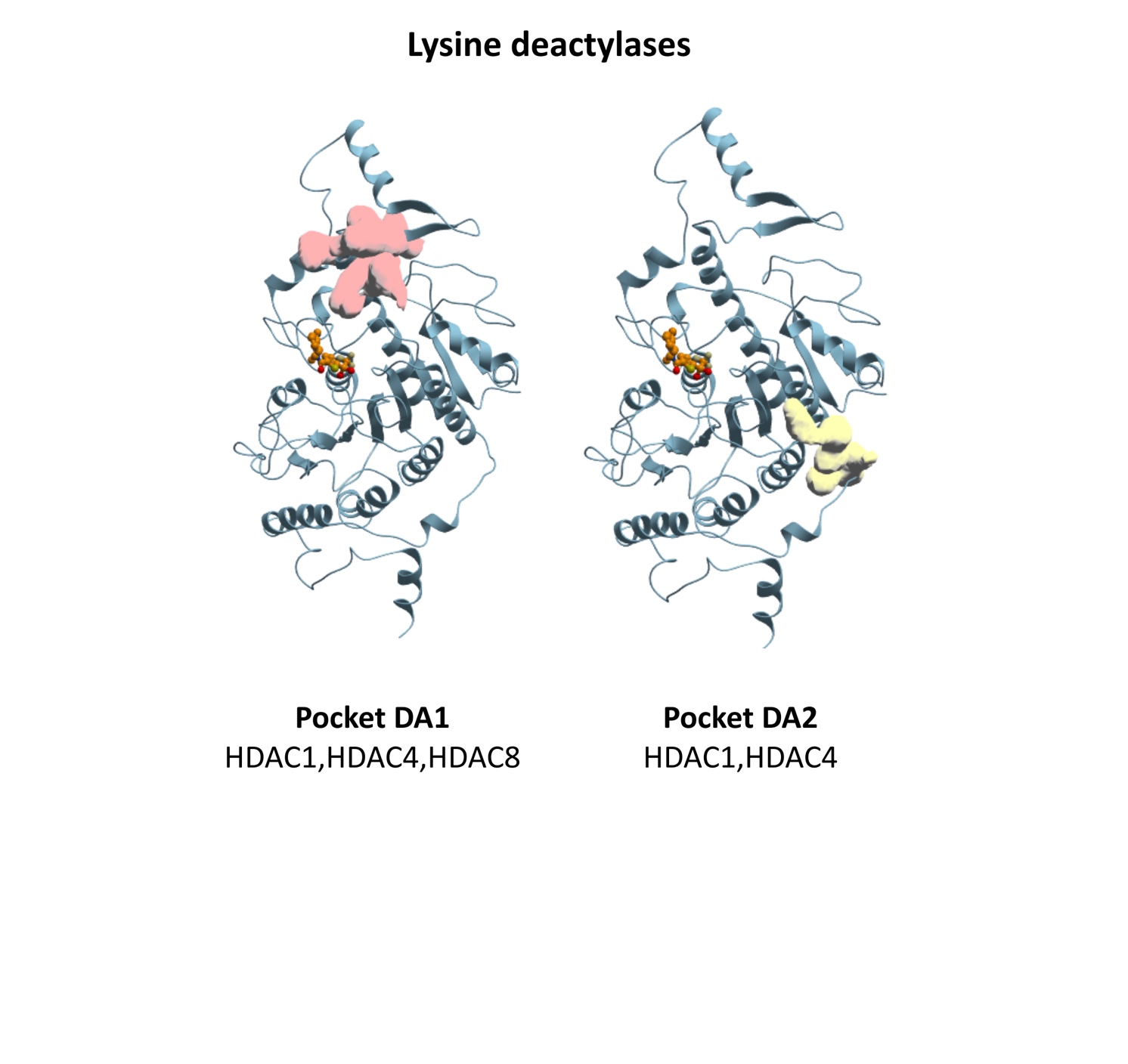


**Figure S7. Recurrent non-catalytic pockets in the catalytic domain of histone deacetylases.** Lysine deacetylase (blue) bound catalytic inhibitor (orange). Reference structure: PDB 2VQJ [22]; reference catalytic inhibitor (orange): PDB 2VQJ [22]. The full list of deacetylases and pockets summarized here is provided in in the database ProxyBind and Table S2.

**
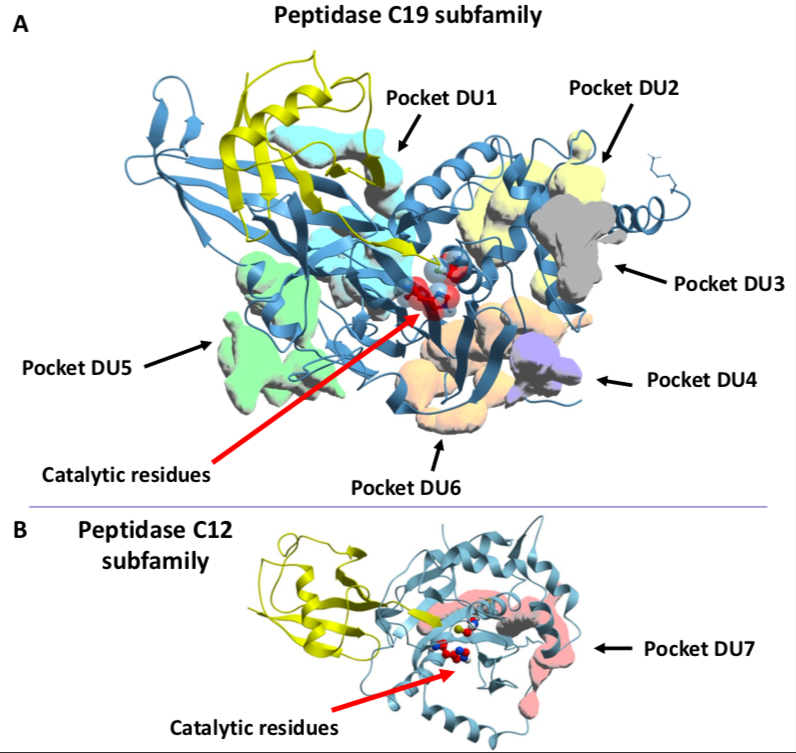
**

**Figure S8. Recurrent non-catalytic pockets in the catalytic domain of deubiquitinases.** A) Peptidase C19-type DUBs: reference structure: USP7 (PDB 1BNF [23]), (blue) bound to ubiquitin (yellow). Reference catalytic inhibitor (orange): PDB 6GH9 [24], B) Peptidase C12-type DUBs: UCHL1 (PDB 3KW5 [25]) (blue) bound to ubiquitin (yellow). Catalytic residues are highlighted in red. The full list of deubiquitinases and pockets summarized here is provided in in the database ProxyBind and Table S2.


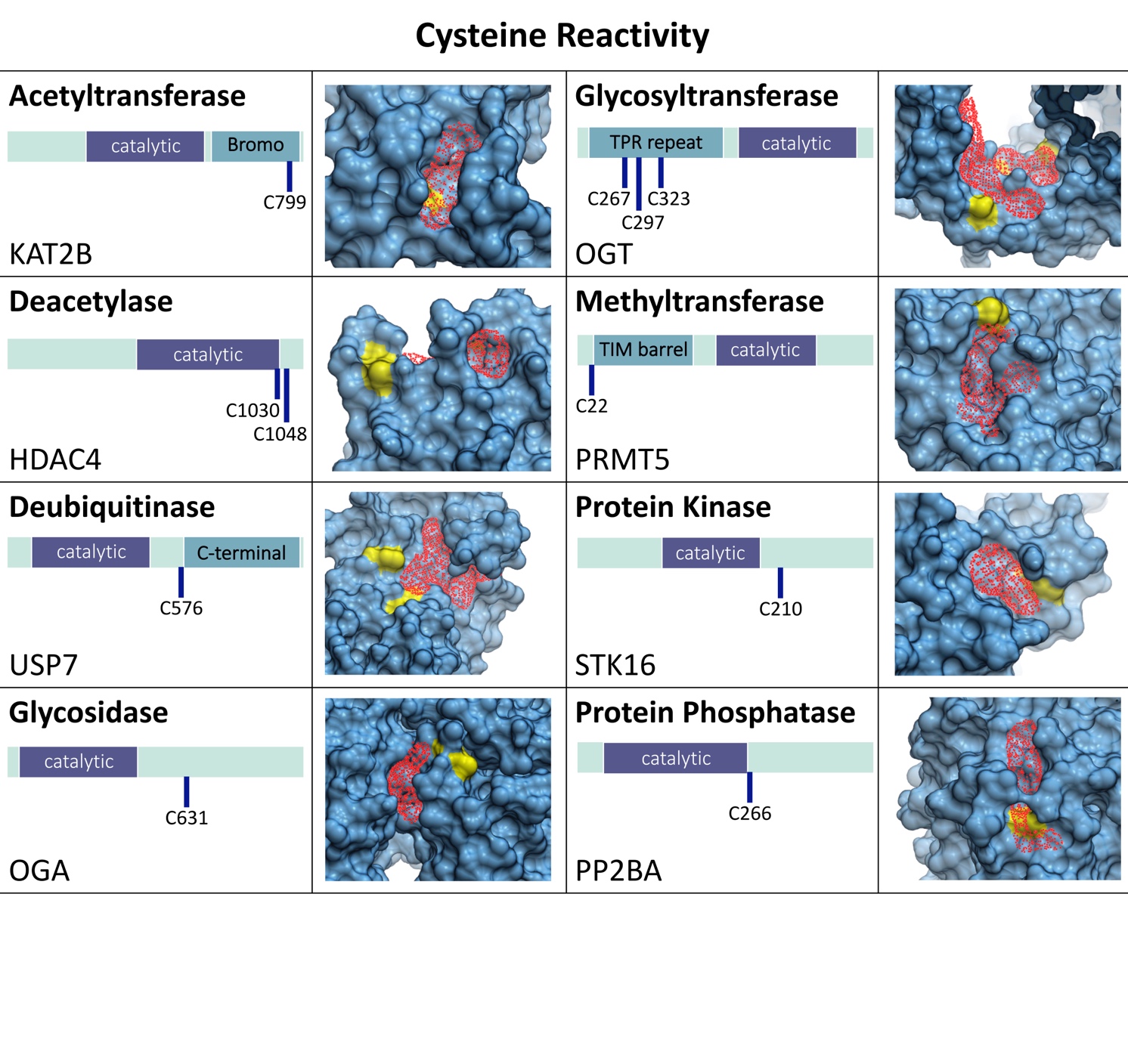


**Figure S9. Examples of non-catalytic pockets with reactive cysteine residue lining the cavity.** Pockets are highlighted in red. Cysteine residues predicted reactive are colored in yellow. Reactive cysteines in the pockets are described in the column ‘Reactive cysteines’ in Table S2 and database.

References

[1] Liu L, Zhen XT, Denton E, Marsden BD, Schapira M. ChromoHub: a data hub for navigators of chromatin-mediated signalling. Bioinformatics 2012;28:2205. https://doi.org/10.1093/BIOINFORMATICS/BTS340.

[2] Liu L, Damerell DR, Koukouflis L, Tong Y, Marsden BD, Schapira M. UbiHub: a data hub for the explorers of ubiquitination pathways. Bioinformatics 2019;35:2882–4. https://doi.org/10.1093/BIOINFORMATICS/BTY1067.

[3] Bateman A, Martin MJ, Orchard S, Magrane M, Agivetova R, Ahmad S, et al. UniProt: the universal protein knowledgebase in 2021. Nucleic Acids Res 2021;49:D480–9. https://doi.org/10.1093/NAR/GKAA1100.

[4] Wang J, Yazdani S, Han A, Schapira M. Structure-based view of the druggable genome. Drug Discov Today 2020;25:561–7. https://doi.org/10.1016/J.DRUDIS.2020.02.006.

[5] Tweedie S, Braschi B, Gray K, Jones TEM, Seal RL, Yates B, et al. Genenames.org: the HGNC and VGNC resources in 2021. Nucleic Acids Res 2021;49:D939–46. https://doi.org/10.1093/NAR/GKAA980.

[6] Mitchell AL, Attwood TK, Babbitt PC, Blum M, Bork P, Bridge A, et al. InterPro in 2019: improving coverage, classification and access to protein sequence annotations. Nucleic Acids Res 2019;47:D351–60. https://doi.org/10.1093/nar/gky1100.

[7] Simpson GL, Bertrand SM, Borthwick JA, Campobasso N, Chabanet J, Chen S, et al. Identification and Optimization of Novel Small c-Abl Kinase Activators Using Fragment and HTS Methodologies. J Med Chem 2019;62:2154–71. https://doi.org/10.1021/ACS.JMEDCHEM.8B01872.

[8] Wood DJ, Lopez-Fernandez JD, Knight LE, Al-Khawaldeh I, Gai C, Lin S, et al. FragLites - Minimal, Halogenated Fragments Displaying Pharmacophore Doublets. An Efficient Approach to Druggability Assessment and Hit Generation. J Med Chem 2019;62:3741–52. https://doi.org/10.1021/ACS.JMEDCHEM.9B00304/SUPPL_FILE/JM9B00304_SI_003.PDF.

[9] Sadowsky JD, Burlingame MA, Wolan DW, McClendon CL, Jacobson MP, Wells JA. Turning a protein kinase on or off from a single allosteric site via disulfide trapping. Proc Natl Acad Sci U S A 2011;108:6056–61. https://doi.org/10.1073/pnas.1102376108.

[10] Perry JJP, Harris RM, Moiani D, Olson AJ, Tainer JA. p38α MAP Kinase C-Terminal Domain Binding Pocket Characterized by Crystallographic and Computational Analyses. J Mol Biol 2009;391:1–11. https://doi.org/10.1016/j.jmb.2009.06.005.

[11] Yan Y, Edward Zhou X, Novick SJ, Shaw SJ, Li Y, Brunzelle JS, et al. Structures of AMP-activated protein kinase bound to novel pharmacological activators in phosphorylated, non-phosphorylated, and nucleotide-free states. J Biol Chem 2019;294:953–67. https://doi.org/10.1074/jbc.RA118.004883.

[12] Li X, Wang L, Zhou XE, Ke J, De Waal PW, Gu X, et al. Structural basis of AMPK regulation by adenine nucleotides and glycogen. Cell Res 2015 251 2014;25:50–66. https://doi.org/10.1038/cr.2014.150.

[13] Mapelli M, Massimiliano L, Crovace C, Seeliger MA, Tsai L-H, Meijer L, et al. Mechanism of CDK5/p25 Binding by CDK Inhibitors. J Med Chem 2005;48:671–9. https://doi.org/10.1021/jm049323m.

[14] Zhang G, Kazanietz MG, Blumberg PM, Hurley JH. Crystal Structure of the Cys2 Activator-Binding Domain of Protein Kinase C8 in Complex with Phorbol Ester. Cell 1995;81:917–24.

[15] Verdaguer N, Corbalan-Garcia S, Ochoa WF, Fita I, Gómez-Fernández JC. Ca2+ bridges the C2 membrane-binding domain of protein kinase Cα directly to phosphatidylserine. EMBO J 1999;18:6329–38. https://doi.org/10.1093/EMBOJ/18.22.6329.

[16] Littler DR, Walker JR, She YM, Finerty PJ, Newman EM, Dhe-Paganon S. Structure of human protein kinase C eta (PKCη) C2 domain and identification of phosphorylation sites. Biochem Biophys Res Commun 2006;349:1182–9. https://doi.org/10.1016/J.BBRC.2006.08.160.

[17] Sharma P, Mahen R, Rossmann M, Stokes JE, Hardwick B, Huggins DJ, et al. A cryptic hydrophobic pocket in the polo-box domain of the polo-like kinase PLK1 regulates substrate recognition and mitotic chromosome segregation. Sci Rep 2019;9. https://doi.org/10.1038/S41598-019-50702-2.

[18] Iversen LF, Andersen HS, Møller KB, Olsen OH, Peters GH, Branner S, et al. Steric Hindrance as a Basis for Structure-Based Design of Selective Inhibitors of Protein-Tyrosine Phosphatases†. Biochemistry 2001;40:14812–20. https://doi.org/10.1021/BI011389L.

[19] Kissinger CR, Parge HE, Knighton DR, Lewis CT, Pelletier LA, Tempczyk A, et al. Crystal structures of human calcineurin and the human FKBP12–FK506–calcineurin complex. Nat 1995 3786557 1995;378:641–4. https://doi.org/10.1038/378641a0.

[20] Xing Y, Xu Y, Chen Y, Jeffrey PD, Chao Y, Lin Z, et al. Structure of Protein Phosphatase 2A Core Enzyme Bound to Tumor-Inducing Toxins. Cell 2006;127:341–53. https://doi.org/10.1016/J.CELL.2006.09.025.

[21] Huhn J, Jeffrey PD, Larsen K, Rundberget T, Rise F, Cox NR, et al. A structural basis for the reduced toxicity of dinophysistoxin-2. Chem Res Toxicol 2009;22:1782–6. https://doi.org/10.1021/TX9001622/SUPPL_FILE/TX9001622_SI_002.QT.

[22] Leonard D, Huang W, Izadmehr S, O’Connor CM, Wiredja DD, Wang Z, et al. Selective PP2A Enhancement through Biased Heterotrimer Stabilization. Cell 2020;181:688-701.e16. https://doi.org/10.1016/j.cell.2020.03.038.

[23] Genera M, Samson D, Raynal B, Haouz A, Baron B, Simenel C, et al. Structural and functional characterization of the PDZ domain of the human phosphatase PTPN3 and its interaction with the human papillomavirus E6 oncoprotein. Sci Rep 2019;9. https://doi.org/10.1038/S41598-019-43932-X.

[24] Pádua RAP, Sun Y, Marko I, Pitsawong W, Stiller JB, Otten R, et al. Mechanism of activating mutations and allosteric drug inhibition of the phosphatase SHP2. Nat Commun 2018 91 2018;9:1–14. https://doi.org/10.1038/s41467-018-06814-w.

[25] Scheufler C, Brinker A, Bourenkov G, Pegoraro S, Moroder L, Bartunik H, et al. Structure of TPR Domain–Peptide Complexes: Critical Elements in the Assembly of the Hsp70–Hsp90 Multichaperone Machine. Cell 2000;101:199–210. https://doi.org/10.1016/S0092-8674(00)80830-2.

[26] Liu F, Li F, Ma A, Dobrovetsky E, Dong A, Gao C, et al. Exploiting an allosteric binding site of PRMT3 yields potent and selective inhibitors. J Med Chem 2013;56:2110–24. https://doi.org/10.1021/JM3018332/SUPPL_FILE/JM3018332_SI_001.PDF.

[27] Bottomley MJ, Lo Surdo P Lo, Di Giovine P Di, Cirillo A, Scarpelli R, Ferrigno F, et al. Structural and functional analysis of the human HDAC4 catalytic domain reveals a regulatory structural zinc-binding domain. J Biol Chem 2008;283:26694–704. https://doi.org/10.1074/JBC.M803514200/ATTACHMENT/AB667A0B-3B61-4516-80E2-B8A1E1714095/MMC1.PDF.

[28] Clarke J, Henrick K, Fersht AR. Disulfide Mutants of Barnase I: Changes in Stability and Structure Assessed by Biophysical Methods and X-ray Crystallography. J Mol Biol 1995;253:493–504. https://doi.org/10.1006/JMBI.1995.0568.

[29] Ward SJ, Gratton HE, Indrayudha P, Michavila C, Mukhopadhyay R, Maurer SK, et al. The structure of the deubiquitinase USP15 reveals a misaligned catalytic triad and an open ubiquitin-binding channel. J Biol Chem 2018;293:17362–74. https://doi.org/10.1074/JBC.RA118.003857/ATTACHMENT/9DA6D65F-EA57-4852-9204-80EFAC55F2C8/MMC1.PDF.

[30] Boudreaux DA, Maiti TK, Davies CW, Das C. Ubiquitin vinyl methyl ester binding orients the misaligned active site of the ubiquitin hydrolase UCHL1 into productive conformation. Proc Natl Acad Sci U S A 2010;107:9117–22. https://doi.org/10.1073/PNAS.0910870107/-/DCSUPPLEMENTAL.
